# Inappropriate Subcutaneous Implantable Cardioverter-defibrillator Shocks—A Rare Case of Triple Counting

**DOI:** 10.19102/icrm.2023.14121

**Published:** 2023-12-15

**Authors:** Sai Nikhila Ghanta, Bader Alotaibi, Hakan Paydak, J. Paul Mounsey, Srikanth Vallurupalli, Subodh Devabhaktuni

**Affiliations:** 1 Department of Internal Medicine, University of Arkansas for Medical Sciences, Little Rock, AR, USA; 2 Department of Medicine, Division of Cardiology, University of Arkansas for Medical Sciences, Little Rock, AR, USA

**Keywords:** Inappropriate shock, subcutaneous ICD, triple counting

## Abstract

Sudden cardiac death (SCD) caused by ventricular tachyarrhythmias is a significant contributor to cardiovascular deaths worldwide. Implantable cardioverter-defibrillators (ICDs) have shown efficacy in preventing and reducing mortality from SCD, but traditional transvenous ICDs have inherent challenges and drawbacks, such as lead fractures, lead-associated endocarditis, and lead failure. To address these issues, subcutaneous ICDs (S-ICDs) have been developed. S-ICDs lack pacing capacity but are a valid alternative for patients at high risk for infection or with difficult venous access. Pre-implantation screening can help prevent inappropriate device shocks. We present a case in which a patient received inappropriate S-ICD therapy, which was attributed to the triple counting of P-, R-, and T-waves in a patient with sinus rhythm. This is an unusual occurrence, and, to the best of our knowledge, there are only a limited number of case reports documenting inappropriate shocks due to the oversensing of P-waves and T-waves.

## Introduction

Sudden cardiac death (SCD) related to ventricular tachyarrhythmias results in >50% of cardiovascular deaths worldwide.^1^ Transvenous implantable cardioverter-defibrillators (ICDs) have shown efficacy in preventing and reducing mortality from SCD.^2,3^ However, traditional ICDs come with inherent challenges and drawbacks of transvenous leads, such as difficulty with venous access, lead fractures, lead-associated endocarditis, damage to valves, cardiac perforation with or without tamponade, and lead failure, in up to 40% of patients.^4^ To minimize some of these limitations, subcutaneous ICD (S-ICD) devices have been developed in an attempt to entirely avoid endovascular access.^5,6^ However, 8.3% of patients with S-ICDs encounter the issue of inappropriate device shocks.^7,8^ Inappropriate shocks are characterized by the delivery of shocks for arrhythmias that are non–life-threatening or due to oversensing.^9^ Oversensing in the context of an S-ICD can be divided into physiological (oversensing of T-waves or double counting of QRS complexes) and non-physiological oversensing (electromagnetic interference or lead fracture).^10^ Among these, T-wave oversensing and low signal amplitude contribute to the majority of inappropriate S-ICD shocks.^11^ While inappropriate shocks due to P-wave oversensing have been routinely reported in hypertrophic cardiomyopathy, attributed to atrial enlargement and higher-amplitude R-waves with a high slew rate,^12,13^ this consideration can be applied to other patient populations. For instance, patients with S-ICDs having low-amplitude QRS complexes may experience T-wave and P-wave oversensing. It is imperative to identify the etiology and prevent inappropriate device shocks due to their significant association with all-cause mortality (hazard ratio [HR], 2.29; *P* = .025).^10^ One such effective approach involves pre-implantation screening using surface electrocardiography (ECG), which can be conducted manually or facilitated by using automated software such as the SMART Pass™ (SP) filter (Boston Scientific Corp., Marlborough, MA, USA).^14,15^ The primary aim of this high-pass filter is to reduce inappropriate shocks by specifically decreasing the oversensing of T-waves. Approval for the SP filter was granted in 2016. This filter can be activated after the device setup process and the selection of the optimal vector. If the sense vector signal meets the minimum QRS amplitude requirements (≥0.5 mV), the device automatically activates the SP filter. The filter itself is a first-order high-pass filter with a corner frequency ranging between 8–9 Hz and a roll-off rate of 20 dB/decade. It primarily reduces signals around the corner frequency while gradually reducing signals at lower frequencies, thus preserving higher-frequency signals (>10 Hz). By considering the fundamental frequency of T-waves (<9 Hz) and QRS complexes (>10 Hz) observed on surface and subcutaneous ECGs, the SP filter is likely to reduce the T-waves while maintaining accurate sensing of QRS complexes due to the improved QRS-to–T-wave ratio. Thenus et al. studied 1984 patients with S-ICDs enrolled in a remote monitoring system over 1 year and calculated the shock incidence for patients with SP enabled or disabled at implantation. They identified that SP reduced the risk of first inappropriate shock by 50% (*P* < .01) and the risk for all inappropriate shocks by 68% (*P* < .001) without any hindrance to appropriate shocks.^16^ The manual screening ECG parameters include QRS duration, corrected QT (QTc) interval, T-wave morphology, and the QRS-to–T-wave ratio obtained in different positions.^14,15^ Studies estimate that screening ECG tools can identify patients at a higher risk of inappropriate shocks with an accuracy of 8%–15%.^15,17^ Other novel screening tools, such as the tube exercise test, can identify patients at risk for inappropriate shocks with positive and negative predictive values of 25% and 100%, respectively.^18^ In our study, we present an unusual case where oversensing of P-waves and T-waves resulted in inappropriate ICD shock.

## Case presentation

**Figure 1:**
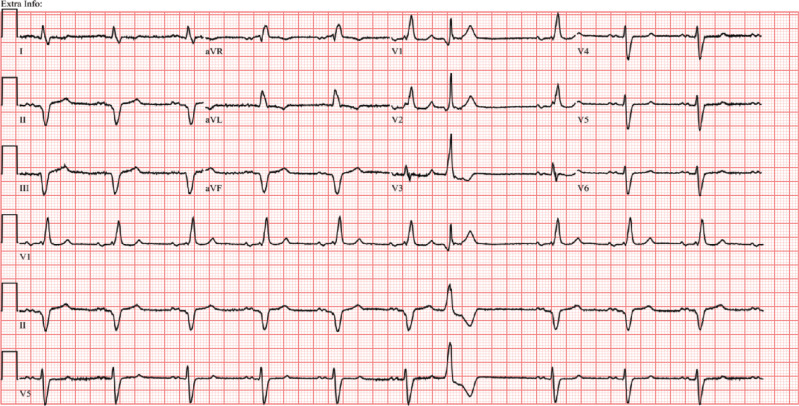
Twelve-lead electrocardiography showing sinus rhythm with occasional premature ventricular complexes and left axis deviation probably due to left anterior fascicular block.

**Figure 2:**
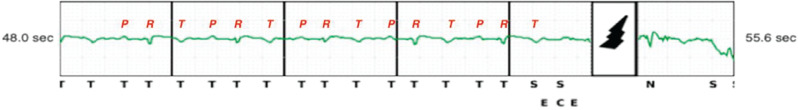
Demonstration of triple counting of P-, R-, and T-waves resulting in inappropriate shock therapy. S denotes sensing and T denotes tachycardia detected.

The patient was a 68-year-old man with a past medical history of peripheral vascular disease status post-amputation with non-ischemic cardiomyopathy, a left ventricular ejection fraction of 20%–25% on guideline-directed medical therapy, and ECG showing sinus rhythm **(Figure 1)**. He received an ICD for the prevention of tachyarrhythmia and SCD.^6,19^ An S-ICD was considered given his history of recurrent lower-extremity stump infection and an elevated risk of bacteremia. Prior to S-ICD implantation, the patient was manually screened in the supine position and was deemed an appropriate candidate in the primary vector only as he failed screening in other vectors for S-ICD implantation. As per the current manufacturer’s guidelines, an S-ICD was implanted given that the patient passed one screening vector. However, the SP filter could not be turned on during the procedure due to small R-waves. A test shock of 10 J was performed, which resulted in ventricular fibrillation (VF), which was successfully defibrillated back to sinus rhythm by reversing the polarity at 80 J with the second shock. One week after device implantation, the SP filter was turned on in the device clinic as R-waves were more prominent. Two weeks later, the patient presented to the emergency department complaining of a jolt that he had felt across his chest. The patient’s S-ICD was interrogated, which confirmed inappropriate shocks due to P- and T-wave oversensing with the SP filter that was disabled automatically prior to the shocks **(Figure 2)**. Due to concerns of inappropriate shocks and after shared decision-making, the S-ICD was removed and a transvenous ICD was implanted instead.

## Discussion

S-ICDs have emerged as an innovative and viable alternative to traditional ICDs, with similar rates of inappropriate shock occurrence.^8^ In light of the benefits of the S-ICD over transvenous ICDs, we selected an S-ICD as the device of choice for our patient. However, the patient experienced inappropriate shocks within weeks of implantation, which was attributed to the triple counting of P-, R-, and T-waves in a patient with sinus rhythm—an uncommon occurrence along with an automatically disabled SP filter. To prevent the undersensing of VF, the SP filter is designed to deactivate when the recorded signal amplitude drops to <0.25 mV (SP-filtered). In our patient, it is likely that he had borderline QRS amplitude, making it easy to deactivate the SP filter automatically. To the best of our knowledge, there are only four case reports documenting inappropriate shocks due to triple counting in patients with S-ICDs.^20–23^ Therefore, we wish to draw attention to a few noteworthy aspects of this case. First, it is important to note that patients who are considered appropriate candidates for S-ICD systems undergo a pre-implantation criteria screening and vector selection for S-ICD systems. This includes modified ECG and the examination of a customized transparent plastic tool against the modified ECG, which assesses the QRS duration, QTc interval, T-wave morphology, and QRS-to–T-wave ratios in different positions. T-wave oversensing has been identified as one of the most common causes of inappropriate shock in S-ICD patients. Therefore, appropriate selection of a sensing vector is crucial to avoid inappropriate S-ICD discharges. This is especially critical in patients who pass the criteria for only a single vector. In our case, as per the current manufacturer’s guidelines, an S-ICD was implanted as the patient passed one screening vector (primary vector), which may not have been sufficient to prevent inappropriate shocks. Further research is needed to determine whether a single pass criterion is adequate for S-ICD implantation or if additional screening criteria are necessary. For instance, in a recently published retrospective observational study conducted by Ben Kilani et al., it was found that the presence of all three vectors, as provided by the automated screening tool, exhibited an independent association with a decreased risk of inappropriate shock (HR, 0.33; 95% confidence interval, 0.11–0.93).^24^ In addition, in a post hoc analysis of data from the Understanding Outcomes with the S-ICD in Primary Prevention Patients with Low Ejection Fraction (UNTOUCHED) trial, conducted by Gold et al., it was observed that there was a tendency toward a lower incidence of inappropriate shocks in patients who had more than one vector available during screening (*P* = .07).^25^ Using data from the S-ICD System Post-approval Study cohort, Burke et al. conducted a proportional hazard analysis and demonstrated that having only one vector available in both the standing and supine positions during screening, as opposed to having two or three validated vectors, was associated with a greater risk of experiencing inappropriate shocks (*P* = .0069).^26^ Future studies should also consider revising the S-ICD screening criteria and re-evaluating the implantation process to minimize the risk of inappropriate shocks.

Another important aspect in consideration is anatomical landmarks: implantation of an S-ICD depends solely on anatomical landmarks (guided with or without fluoroscopy), which determine the sensing vectors. There are currently no recommendations concerning screening patient anatomy prior to S-ICD implantation. However, modifying and improving upon available screening tools is appropriate and would be of value on a case-by-case basis. There are three sensing vectors by which the S-ICD system can recognize arrhythmias. The system has two electrodes: a distal electrode and a proximal electrode. The primary vector records ECG from the proximal electrode to the can, the secondary vector records ECG from the distal electrode to the can, and the alternative vector records the ECG vertically from the distal electrode to the proximal electrode.

Our patient passed the S-ICD screening with only one vector (primary) in both the supine and sitting positions; this was deemed sufficient as per the current guidelines for S-ICD system implantation.

**Figure 3:**
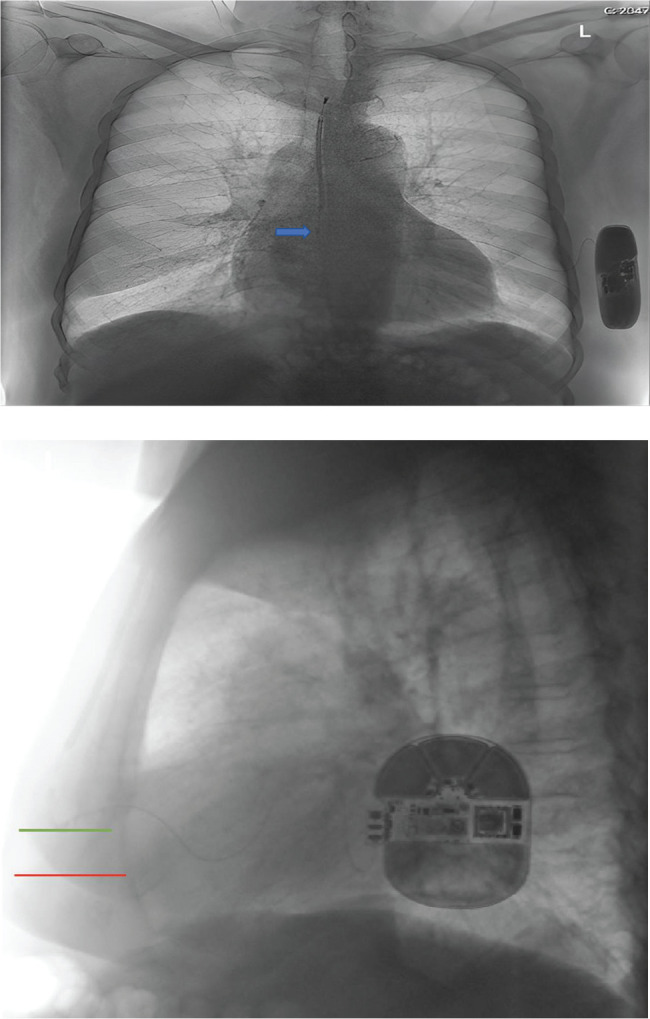
Chest X-ray (posteroanterior and lateral views). The blue arrow indicates the location of the ring electrode in the posteroanterior view. The red line indicates the tip of xiphisternum, and the green line indicates the xiphoid incision level at the appropriate location in the lateral view.

Implantation of the device involves marking the anatomical landmarks and testing the system in the electrophysiology laboratory with the patient in the supine position. However, misalignment between the xiphoid incision, which approximates the proximal electrode location, and the heart may occur **(Figure 3)**, potentially leading to the oversensing of P-waves due to the proximity of the electrode to the right atrium. We speculate that this was the most likely reason for the oversensing of P-waves in our patient. This highlights an important aspect to consider while implanting this device and to note the location of the proximal electrode to the right atrium and the right ventricle, especially if only the primary vector pass criteria are met. Adjusting the subxiphoid incision site could be a potential option to minimize inappropriate shocks in this scenario. Due to these shortcomings, inappropriate shock was delivered due to triple counting despite a sinus rhythm.

In conclusion, our case highlights the limitations of the current prescreening recommendations and current implantation technique. To overcome these limitations, larger studies or more cases should be published to address these issues at hand.
